# Supplementary material for: Modulating Cholesterol Metabolism via ACAT1 Knockdown Enhances Anti-B-Cell Lymphoma Activities of CD19-Specific Chimeric Antigen Receptor T Cells by Improving the Cell Activation and Proliferation
Source: Cells. 2024 Mar 21;13(6):555. doi: 10.3390/cells13060555 (PMC10969720; doi:10.3390/cells13060555)
Supplement: Supplementary file 1 [file cells-13-00555-s001.zip › cells-2857616-supplementary.pdf]

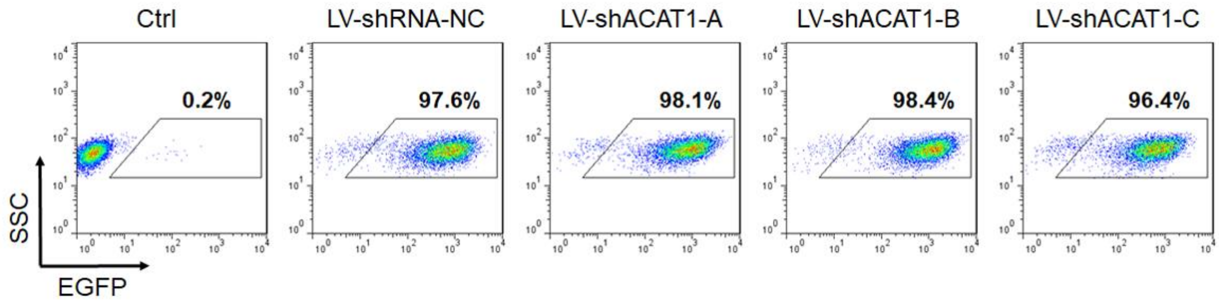

Supplementary Figure 1. Analysis of transduction efficiency of lentivirus expressing ACAT1-shRNAs. Jurkat cells were transfected with lentivirus expressing ACAT1-shRNAs in MOI of 5 and EGFP-positive cells were detected by flow cytometry.

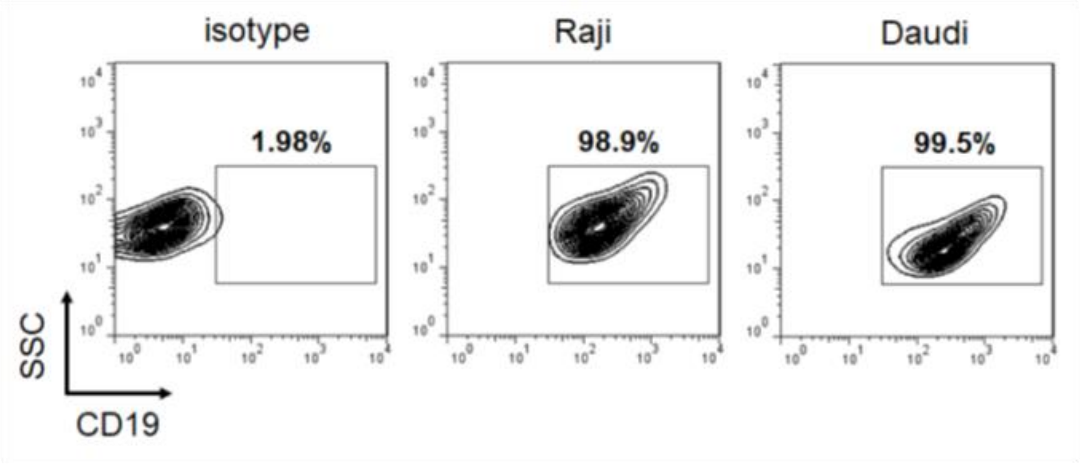

Supplementary Figure 2. CD19 expression on Raji and Daudi cell lines. The expression was examined by flow cytometry using fluorescently labeled anti-human CD19 antibodies.
